# Supplementary material for: Deciphering the Genomic Landscape and Virulence Mechanisms of the Wheat Powdery Mildew Pathogen Blumeria graminis f. sp. tritici Wtn1: Insights from Integrated Genome Assembly and Conidial Transcriptomics
Source: J Fungi (Basel). 2024 Apr 3;10(4):267. doi: 10.3390/jof10040267 (PMC11051031; doi:10.3390/jof10040267)
Supplement: Supplementary file 1 [file jof-10-00267-s001.zip › Supplementary_Figures_S1-S5.pdf]

Supplementary Figures

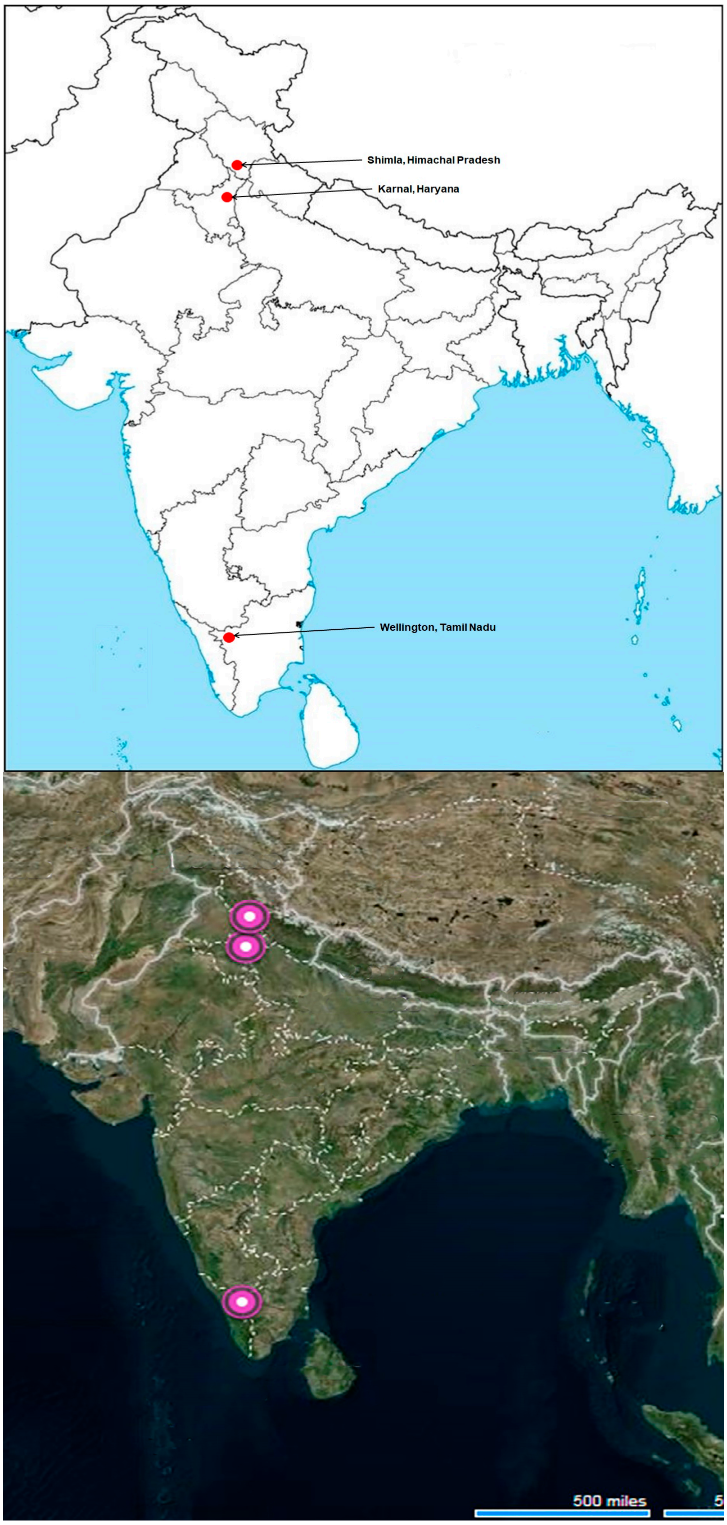

Supplementary Figure S1. Sampling sites in India for *Blumeria graminis* f. sp. *tritici* Wtn1 inciting powdery mildew in wheat; the sites represent wheat growing belt in India

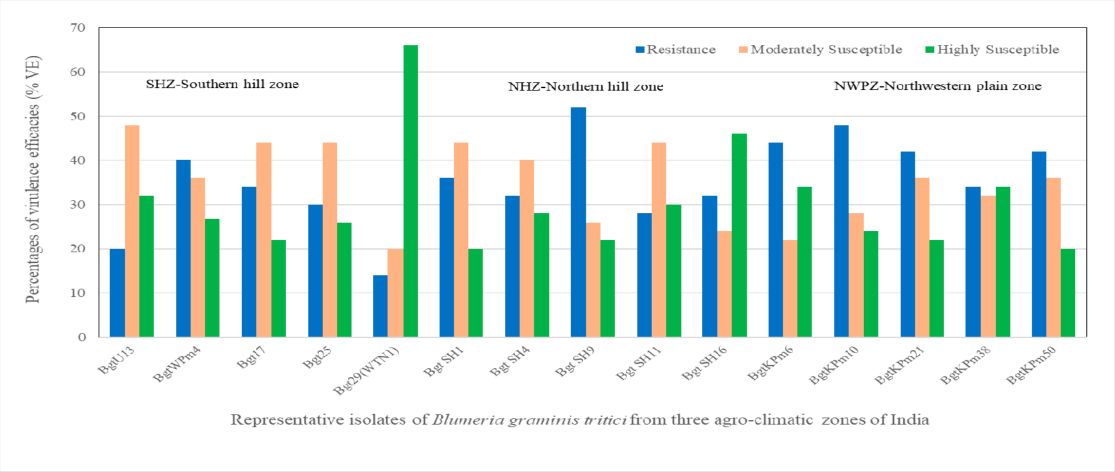

Supplementary Figure S2. Assessment of Virulence Frequencies (VF) of *Blumeria graminis* f. sp. *tritici* Isolates from Diverse Agroclimatic Zones on Indian Wheat Cultivars, and Near Isogenic Lines Carrying Pm Genes Under Controlled Conditions. The bars in the data illustrate the cumulative virulence profile (%) across all *Blumeria graminis* f. sp. *tritici* isolates. Out of the 15 isolates tested, *Blumeria graminis* f. sp. *tritici* 29 (*Blumeria graminis* f. sp. *tritici*_Wtn1) from SHZ (Wellington) exhibited the highest virulence frequency at 66%, followed by *Blumeria graminis* f. sp. *tritici* SH16 at 46%, and *Blumeria graminis* f. sp. *tritici* KPm10 and *Blumeria graminis* f. sp. *tritici* KPm38 at 34%.

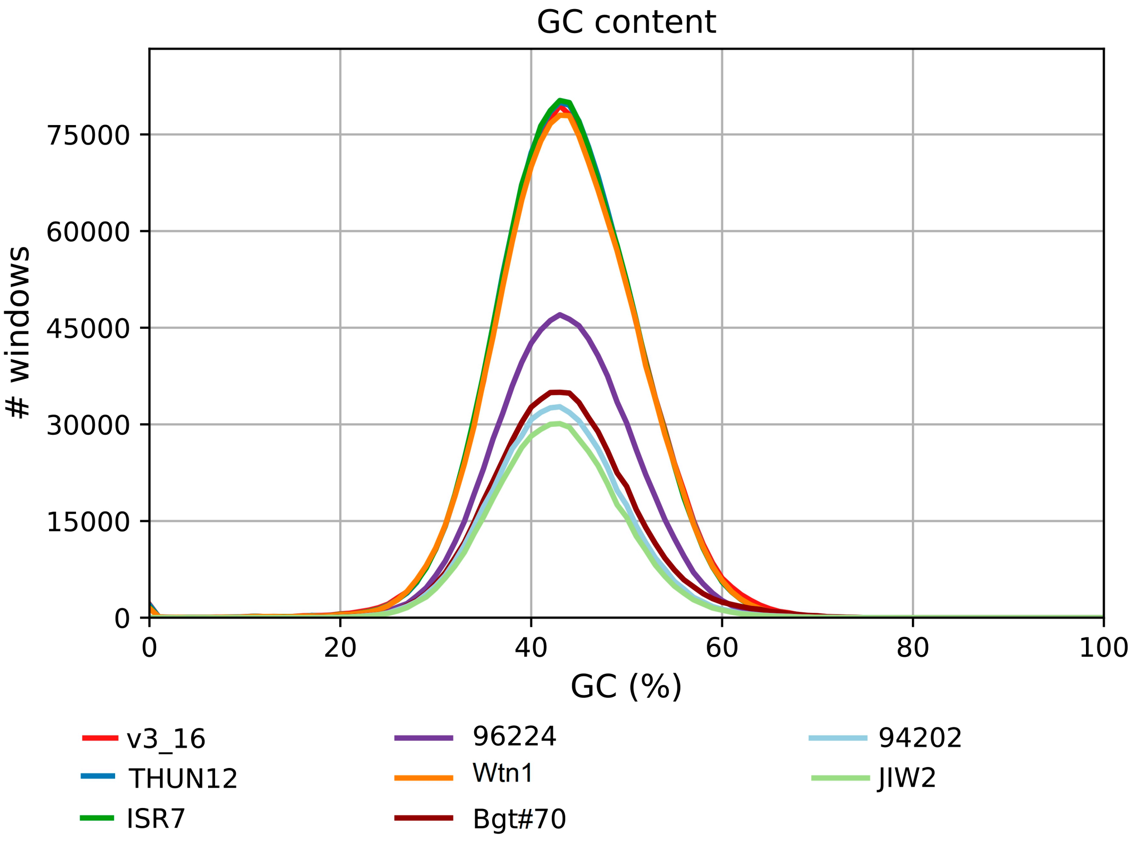

Supplementary Figure S3. GC content assessment of *B. graminis* genomes using QUAST.

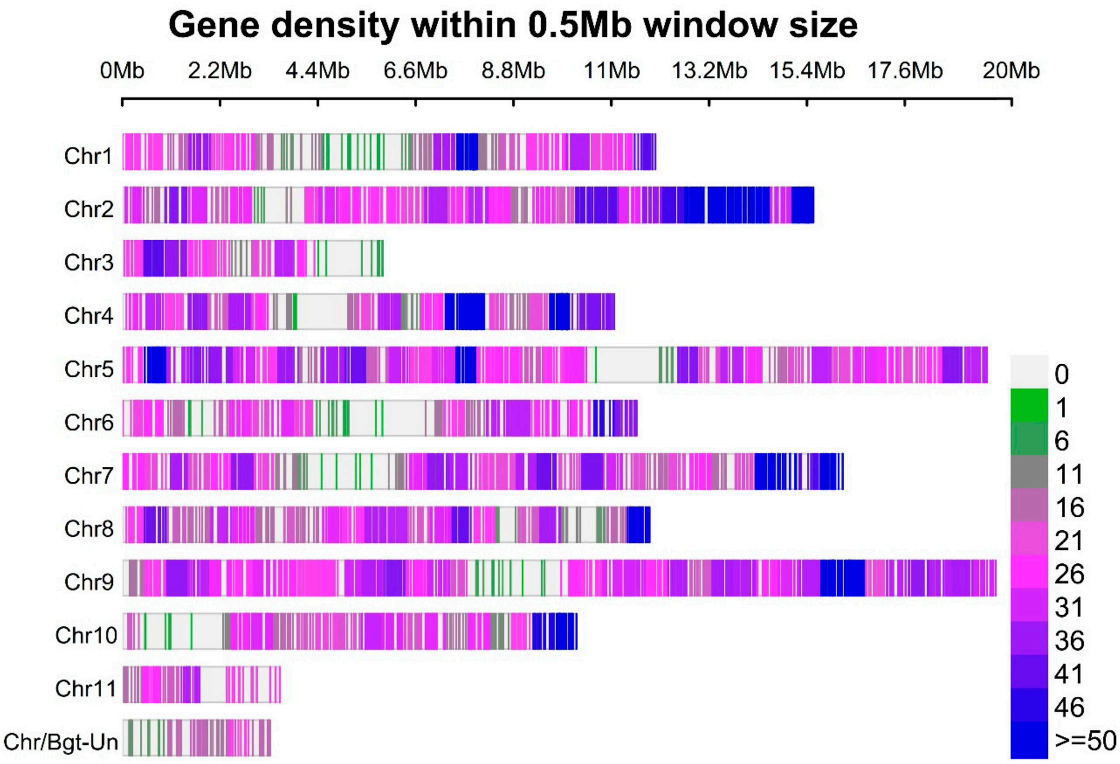

Supplementary Figure S4. The identified gene density plot with respect to chromosome wise representing number of gene density within 0.5 Mb window size, in *Blumeria graminis* f. sp. *tritici* Wtn1 genome.

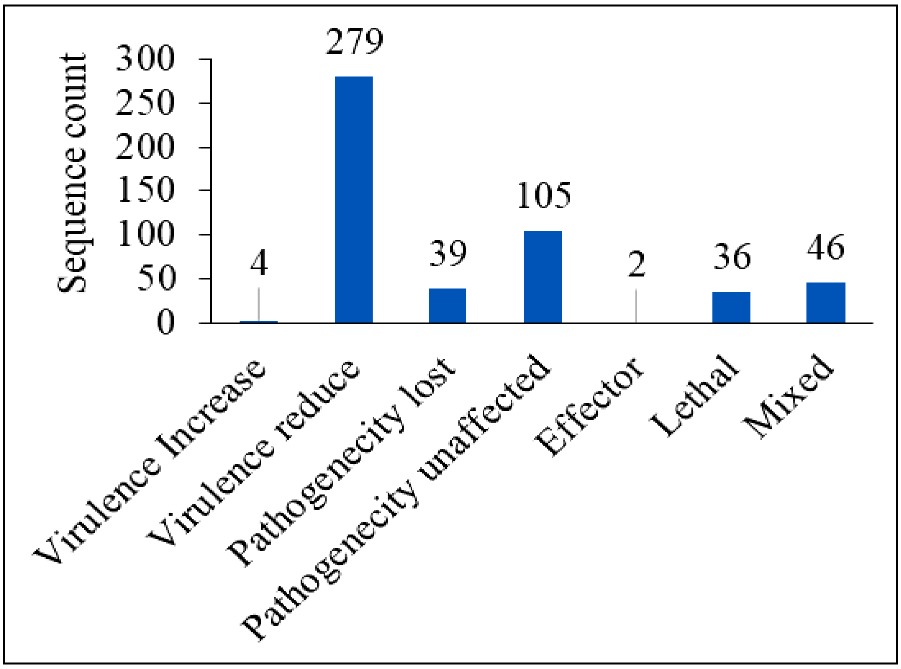

Supplementary Figure S5. Genomic features of pathogenicity and virulence factors in *Blumeria graminis* f. sp. *tritici* Wtn1 deciphered using Pathogen-Host Interaction (PHI) database.
